# Rectovaginal Fistula as a Complication of Fecal Management System

**DOI:** 10.1177/2324709619869368

**Published:** 2019-08-17

**Authors:** Emily Butts, Sandeep Anand Padala, Anusha Vakiti, Vamsi Kota

**Affiliations:** 1 Augusta University Medical Center, Medical College of Georgia, Augusta, GA, USA

**Keywords:** fecal management system, intrarectal catheter, rectovaginal fistula, veno-occlusive disease, fecal incontinence

## Abstract

We report a rare complication of the use of an intrarectal catheter. An 18-year-old female with T-cell acute lymphoblastic leukemia post-matched unrelated donor allogeneic stem cell transplantation (auto-SCT) developed hepatic encephalopathy secondary to hepatic sinusoidal obstructive disease. A fecal management system was used to contain and divert fecal matter in this immobilized patient. Approximately 1 month after placement of an intrarectal catheter, stool was noted in the vaginal vault. Speculum examination confirmed development of a rectovaginal fistula.

## Introduction

Fecal management systems (FMS) are used to contain and divert liquid or semiliquid fecal material in critically ill, bed ridden, or immobilized patients with impaired bowel control. FMS consists of a stool collection bag attached to a flexible catheter with a soft balloon to create a seal when inserted into the distal rectum. It is designed to prevent mucosal damage as it is small and soft. A balloon is then inflated to keep the catheter in place. Benefits of FMS include protecting skin integrity, preventing transmission of nosocomial infections such as *Clostridium difficile*, and improving the ease of managing incontinence for nursing staff and caregivers.^1^ These devices are generally considered safe with minor complications reported including over-inflation of the balloon, temporary anal atony, and excessive leak of stool around the device. In a retrospective review of 50 patients, there were no reported major adverse events such as the formation of fistula or mucosal necrosis.^2^ Few episodes of gastrointestinal bleeding related to use of FMS have been reported in the literature.^3-5^ In this article, we report a case of rectovaginal fistula (RVF) that developed in a critically ill 18-year-old female who had FMS for 31 days.

## Case

An 18-year-old, Caucasian female with T-cell acute lymphoblastic leukemia developed hepatic sinusoidal obstructive syndrome as a complication of matched unrelated donor transplant (hematopoietic stem cell transplant [HSCT]). The patient was treated with preconditioning therapy of cyclophosphamide and total body irradiation prior to HSCT approximately 4 months after diagnosis. Approximately 2 weeks after HSCT, she became critically ill with early-onset veno-occlusive disease. She developed severe veno-occlusive disease with multi-organ failure including hepatic encephalopathy, acute kidney injury requiring hemodialysis, and respiratory failure requiring mechanical ventilation. In addition to defibrotide, rifaximin and lactulose were started to manage the hepatic encephalopathy, and FMS was used to contain and divert liquid stool. Approximately 1 month after placement of FMS, examination noted stool in the vaginal vault. Speculum examination and bimanual examination revealed approximately 2 cm opening in the posterior wall of the vagina over the rectum. The opening of the rectal tube was palpated within the vagina, for which obstetrics-gynecology was consulted and recommended likely surgical repair due to the size of the fistula once patient was stable for surgery. The FMS was kept in place in order to decrease stool burden in the area. Two days after initial identification of the RVF, the patient expired after becoming acutely hypoxic and hypercapnic resulting in pulseless electrical activity arrest, presumed to be from septic shock.

## Discussion

Fecal incontinence is a common problem in critically ill patients. There is always an increased risk of nosocomial infections and skin-related complications in the critical care setting or in patients with chronic comorbid conditions and those who are immobilized.^1^ To better serve these groups, careful attention needs to be paid with regard to their fecal management system as it tends to be ignored routinely. FMS not only helps in controlling the infection rates and protecting the integrity of the perineal skin, but also promotes healing of the wounds or pressure ulcers. It also has a significant impact financially, as it would greatly cut down the frequent linen changes and staffing issues. Incontinence not only causes discomfort but also hugely affects the dignity or self-esteem of a patient.^1,2^ Despite FMS being generally considered a safe method of diverting and collecting fecal matter in critically ill or bed-bound patients, there are limited randomized, controlled trials evaluating the safety and effectiveness of these systems.

Whiteley et al conducted a retrospective review in the acute care patients and found that the complication rates for longer duration, which is 17+ days, was significantly higher. Majority of the study group had the catheter for less than 29 days and no serious adverse events such as mucosal necrosis or fistula were noted. The severity of the anal atony was related to the duration of the catheter placement.^2^ Ousey et al conducted a review of the literature about current FMS available for management of acute fecal incontinence in hospitalized patients. Primary outcomes of the 10 included studies reviewed were safety of FMS and infection control. There was a single reported case of rectal bleeding in a patient receiving antithrombotic agents. Additionally, there was some evidence to support the reduction in cross-contamination by preventing the need for frequent bed linen changes.^6^ A’Court et al reported a rectourethral fistula following FMS after 18 days of placement.^7^

A RVF is an abnormal connection starting from the rectal or the anal epithelium extending into the vagina. It can be congenital or acquired. Acquired RVFs result from an underlying surgery, obstetric injury, prolonged labor, pelvic diseases, inflammatory bowel diseases, and malignancies. The most common being traumatic obstetric injury such as third- and fourth-degree lacerations during vaginal birth.^8^ Gastrointestinal pathology including diverticular disease, inflammatory bowel disease (more common with Crohn’s disease due to transmural involvement), and malignancies have also been reported.^9^ Finally, radiation can result in a delayed development of RVF due to chronic tissue inflammation.^10^ The incidence can go high in people with underlying history of hysterectomy and high-dose radiation treatment. Depending on the size of the fistula, it can be classified into small (<2.5 cm) and large (>2.5 cm) in diameter. The development of RVF in our patient with FMS is to our knowledge the first reported in the literature. Due to hemodynamic instability, imaging could not be done to assess the exact size of the fistula.

It is important to note that our patient did receive preconditioning therapy with cyclophosphamide and total body irradiation prior to HSCT. RVF has been a reported complication of patients receiving pelvic radiotherapy for treatment of cervical cancer. However, the average interval from initiation of radiation to fistula formation was 22 months, ranging from 3 months to 12 years.^11^ In the case of our patient, the RVF was identified less than 2 months from receiving total body irradiation. Therefore, in our patient’s case, we feel that the development of the RVF likely resulted from mucosal breakdown secondary to long-term use of FMS, in approximately 31 days. Given the severity of her illness and ongoing diarrhea, she remained on FMS beyond 29 days and was replaced once. The repeat blood cultures did not reveal any growth, which could be attributed to her being on multiple antibiotics for ongoing septic shock. It could be postulated that, as the duration of intrarectal catheter increases, it might lead to serious life-threatening complications, which have not been studied or reported yet. The development of this complication highlights the importance of proper digital rectal examination prior to insertion of an intrarectal catheter to examine for any contraindications including mucosal damage, strictures, or fistulas. Additionally, it is important to examine the patient regularly for development of fistula, as this is a complication associated with significant morbidity. In our patient’s case, obstetrics-gynecology recommended surgical repair of the fistula due to the size. However, due to her critical condition, she expired prior to becoming stable for surgery.

## Conclusion

This case presents a novel complication of FMS and highlights the importance of careful observance of FMS for complications including RVF. The studies have shown that the rates of complications were higher when used beyond 17 days. We strongly feel that the patients need to be examined regularly and the catheter be discontinued as soon as possible. It could be postulated that, as the duration increases, it might lead to serious complications, which have not been studied or reported yet.
